# Advancing Age and the rs6265 BDNF SNP are Permissive to Graft-induced Dyskinesias in Parkinsonian Rats

**DOI:** 10.21203/rs.3.rs-4243843/v1

**Published:** 2024-05-02

**Authors:** Kathy Steece-Collier, Natosha Mercado, Carlye Szarowicz, Jennifer Stancati, Caryl Sortwell, Samuel Boezwinkle, Timothy Collier, Margaret Caulfield

## Abstract

The rs6265 single nucleotide polymorphism (SNP) in the gene for brain-derived neurotrophic factor is a common variant that alters therapeutic outcomes for individuals with Parkinson’s disease (PD). We previously investigated the effects of this SNP on the experimental therapeutic approach of neural grafting, demonstrating that young adult parkinsonian rats carrying the variant Met allele exhibited enhanced graft function compared to wild-type rats, and also exclusively developed aberrant graft-induced dyskinesias (GID). Aging is the primary risk factor for PD and reduces graft efficacy. Here we investigated whether aging interacts with this SNP to further alter cell transplantation outcomes. We hypothesized that aging would dampen enhancement of graft function associated with this genetic variant and exacerbate GID in all grafted subjects. Unexpectedly, beneficial graft function was maintained in aged rs6265 subjects. However, aging was permissive to GID induction, regardless of genotype, with the greatest incidence and severity found in rs6265 expressing animals.

**Author contributions:** KSC, NMM, TJC, CES designed experiments; KSC acquired funding. KSC, TJC, CES carried out stereotaxic lesioning and cell grafting surgeries. NMM, KSC carried out behavioral assays and data collection. NMM, JAS, SAB, CAS, MEC carried out post-mortem experimental assays and data collection. NMM, CAS, MEC performed statistical analysis and graphing of data. NMM, KSC, CAS, MEC contributed to data interpretation. NMM, CAS, KSC, MEC composed figures and performed primary manuscript writing and assembly. All authors provided intellectual input and editing of this manuscript.

## INTRODUCTION

Parkinson's disease (PD) is a relentlessly progressive neurodegenerative disorder estimated to impact greater than 10 million people worldwide. Despite numerous advances in therapeutic options for PD, individual responses remain highly variable. Even the dopamine (DA) precursor levodopa, considered to be the gold-standard for treatment of motor dysfunction and used for over 50 years, elicits a wide range of efficacy [1, 2]. Additionally, levodopa-induced dyskinesia (LID), the common side effect of long-term levodopa therapy is estimated to occur in up to 90% of individuals with PD within 10 years of treatment [3-5]. Accordingly, efforts continue to identify additional therapeutic options for PD.

For more than three decades, therapeutic grafting of replacement DA neurons has been implemented to restore lost DA terminals within the striatum [6-10]. Engraftment of fetal ventral mesencephalic (VM) DA neurons, has shown significant therapeutic benefit, albeit limited to only a subpopulation of individuals with PD [8, 11-14]. While clinical trials have been executed carefully, skepticism remains due to lack of consistent benefit and the troubling development of the graft-derived side effect known as graft-induced dyskinesia (GID) [8]. Indeed, it was the expression of GID in subpopulations of graft recipients that led to a worldwide moratorium in 2003, halting clinical trials of cell-replacement therapies for PD [9, 10, 15]. Following the resolution of the moratorium, several clinical grafting studies are now ongoing or planned (e.g., [9], clinical trial identifiers NCT04802733, NCT01898390,

NCT03309514, NCT03119636, NCT04146519). While continuing iterative studies have increased understanding of the role of global factors (e.g., disease severity, host age, etc.) in the outcomes of neural transplantation in PD, the role of specific genetic variations remained entirely unexplored until a recent study undertaken by our group that focused on the brain-derived neurotrophic factor (BDNF) single nucleotide polymorphism (SNP), rs6265 [16].

One approach to understanding the inherent heterogeneity of PD is to study genetic risk factors that might impact therapeutic efficacy. Here we focus on the rs6265 SNP (Val66Met, aka: Val/Met) that occurs in the *Bdnf* gene. Both the heterozygous major allele (Val/Met) and homozygous minor allele (Met/Met) of rs6265 result in decreased activity-dependent release of BDNF by disrupting packaging and transport of the protein, thereby resulting in a significant decrease in available BDNF in carriers of this SNP [17, 18]. While the rs6265 SNP is not thought to be associated with PD incidence [19-22], it has been documented to alter therapeutic responses of individuals with PD to levodopa [23, 24]. This SNP has a high, but heterogenous, global prevalence of approximately 20% (e.g., 22% in Caucasian populations, 72% in Asian populations, 2% African American [25-27]). Considering the prevalence of the rs6265 SNP and the influential roles of BDNF in the grafted brain (e.g., supporting embryonic VM graft survival and function [28-33] and promoting dendritic spine growth and formation of synapses [34] important to striatal graft-host integration), we hypothesized that this genetic risk factor may underlie the variability in clinical response to DA neuron grafting in individuals with PD.

Previously, we reported a paradoxical enhancement of graft-derived neurite outgrowth and functional benefit in young adult parkinsonian rats (8 mos at time of lesioning) homozygous for the Met allele, engrafted with wild-type (WT) embryonic VM DA neurons, as compared to WT grafts in WT hosts [16]. Compellingly, the homozygous SNP rats also uniquely developed aberrant GID behavior, in contrast to the absence of GID in WT subjects engrafted with WT DA neurons [16].

Acknowledging the prevalence of PD in the aged population and the negative impacts of aging on efficacy of neural grafting ([35, 36]), we have expanded our investigations into late middle-aged rats (15 mos at time of lesion) to provide important translational insight into whether advancing age interacts with the common human rs6265 SNP to further alter grafted DA neuron function and/or dysfunction. We hypothesized that advancing age would diminish the enhancement of graft function associated with this genetic variant while also exacerbating GID in all grafted subjects regardless of genotype. We report here that counter to the first part of our hypothesis, behavioral enhancement associated with rs6265 was maintained with advancing age, demonstrated by a significant acceleration in the reduction of LID severity post-grafting in parkinsonian rats homozygous for rs6265 compared to parkinsonian WT rats. However, in keeping with the latter part of our hypothesis, the current data demonstrate that advancing age is permissive to the induction of GID in subjects of both genotypes, although Met/Met rats showed a higher incidence and significantly more severe GID. Collectively, these preclinical findings provide evidence suggesting that both host genotype and age contribute, and synergize, to impact GID expression. Understanding how to manage these variables, common to patients with PD, should be considered as clinical trials continue to allow for optimal therapeutic outcome following DA neuron replacement strategies.

## MATERIALS AND METHODS

The experimental design and timeline are shown in **Figure 1**.

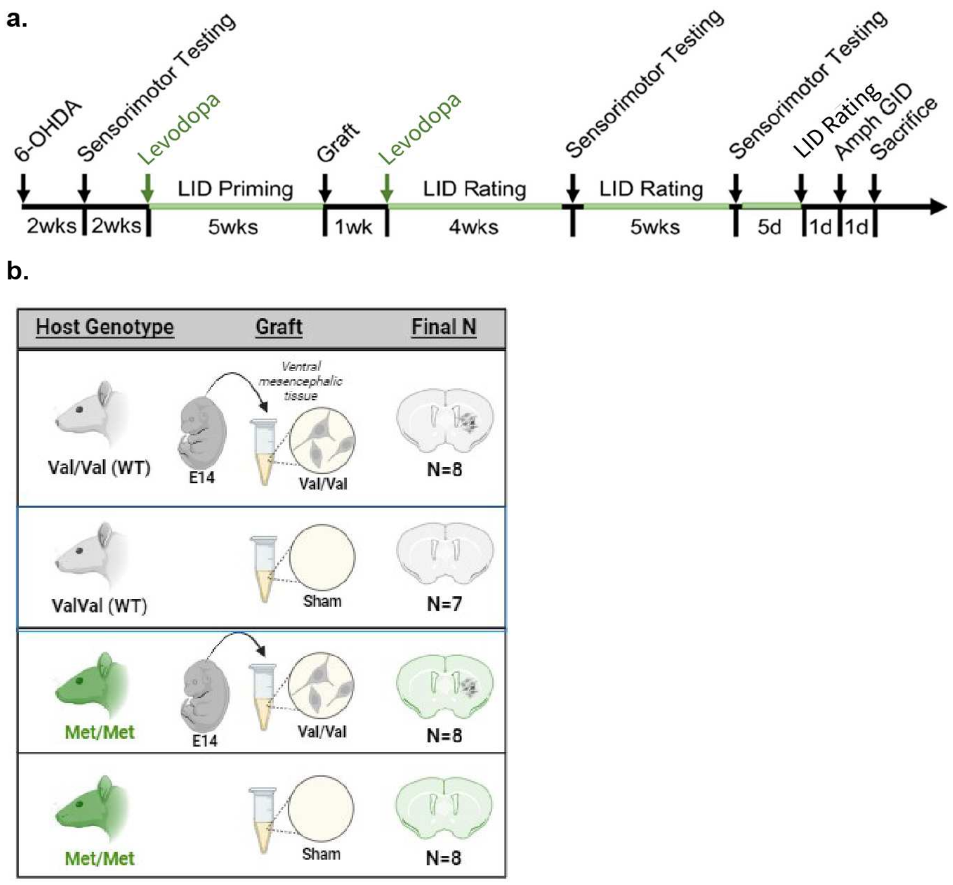

**Figure 1. Experimental Overview. (a) Experimental timeline** of surgical procedures, drug administration, and behavioral evaluation. Rats were rendered unilaterally parkinsonian via stereotaxic injection of 6-hydroxydopamine (6-OHDA) into the substantia nigra and medial forebrain bundle. Two weeks later, drug-free sensorimotor behavior was evaluated using the "drag" and the "sticky paws" tests to assess lesion status and collect baseline behavioral data as a secondary measure of graft efficacy (**Suppl Figure 1**). Two weeks after sensorimotor testing, rats began priming with daily levodopa to induce stable levodopa-induced dyskinesias (LID). After 5 weeks of levodopa priming, all rats received an intrastriatal graft of ventral mesencephalic (VM) dopamine (DA) neurons or a cell-free sham graft. VM tissue was dissected from Val/Val embryonic day 14 Sprague-Dawley rat pups, dissociated, then transplanted into middle-aged Val/Val (WT) and Met/Met rats (**b**). Levodopa was withdrawn for one week following graft surgery after which levodopa treatment was reinitiated. LID, which served as the primary measure of graft efficacy, were evaluated for 10 weeks following DA neuron grafting. Drug-free sensorimotor behaviors were assessed at 5- and 10-weeks post-grafting as a measure of "OFF-time" graft function. As an indicator of graft dysfunction, amphetamine-mediated graft-induced dyskinesias (Amph GID) were evaluated 24 hours following the final LID assessment. Rats were sacrificed 24 hours following GID evaluation. **(b) Experimental design** schematic depicting the experimental groups and final group size upon completion of the study.

## Experimental Subjects

Adult male Sprague-Dawley rats homozygous for the variant Met allele (14-15 mos at lesioning; 19-20 mos at sacrifice) were obtained from our in-house colony derived from CRISPR knock-in rats carrying the valine to methionine polymorphism (Val68Met, Val/Met) in the rat *Bdnf* gene (CD® International Genetic Standardization Program, Charles River Laboratories, Wilmington, MA, USA) as detailed previously [16]. Briefly, rats carrying the valine to methionine polymorphism (Val/Met) in the rat *Bdnf* gene (GenBank: NM_001270630; Ensembl: ENSRNOG00000047466) were generated by Cyagen Biosciences (Santa Clara, CA) using CRISPR/Cas-mediated homologous recombination. Rats have two additional threonine amino acids at codon 57 and 58, making the rat Val68Met equivalent to the human Val66Met SNP. There is 96.8% homology between the rat *Bdnf* gene and the human *BDNF* gene (BLAST queries: P23560 and P23363). WT rats from the same genetic background were purchased at 6 months of age (N=27 Met/Met, N=24 WT) (CD® International Genetic Standardization Program, Charles River Laboratories) and aged in-house for 6 months before experimentation. The age of the experimental subjects at lesioning is considered 'middle-age', however, by the end of the experiment they are considered 'aged' ($\geq$18 mo) [37, 38]; for simplicity sake, we refer to them as middle-age in this manuscript. All experimental procedures were approved by the Michigan State University Institutional Animal Care & Use Committee.

A small number of Met/Met rats (N=3) were humanely euthanized due to illness and rapid weight loss presumed to be a result of spontaneous pituitary adenomas, which were observed postmortem and are common in aging rats [39, 40]. Additional rats were removed from the study due to deaths unrelated to the experiment (N=8), while others were excluded *a priori* (i.e., prior to grafting) because they did not develop LID (N=8; indeed, a small percentage of outbred Sprague-Dawley rats are typically LID-resistant [41]). One Met/Met rat was excluded from postmortem analyses due to severe deformity/compression of the brain resulting from a large pituitary adenoma. Final experimental cohorts were N=16 Met/Met rats and N=15 WT rats (**Figure 1b**).

## Nigrostriatal Lesioning

Rats were rendered unilaterally parkinsonian via stereotaxic injection of 6-hydroxydopamine (6-OHDA) per our usual protocol [16, 42]. Briefly, rats were anesthetized with inhalant isoflurane (2-3%; Sigma, St. Louis, MO, USA) and secured in a stereotaxic frame (David Kopf Instruments, Tujunga,

CA, USA). Each rat received a total of 4 µl of 6-OHDA (5 mg/ml) using a 5 µl Hamilton syringe with a 26-gauge needle divided equally between two midbrain sites (2 µl at each site; 0.5 µl/min): the substantia nigra (SN; 4.8 mm posterior, 1.7 mm lateral, 8.0 mm ventral, relative to bregma) and the medial forebrain bundle (MFB; 4.3 mm posterior, 1.6 mm lateral, 8.4 mm ventral, relative to bregma). Immediately following surgeries, rats received the analgesic carprofen (as Rimadyl®; 5 mg/kg).

**Pre-Graft Lesion Confirmation**

We employed two drug-free tests to evaluate lesion status and to provide a secondary measure of graft function [43-45] in our aging parkinsonian rats. These tests, referred to here as the “drag” and the “sticky paws” tests, were assessed 2 weeks post-lesioning/ 7 weeks pre-graft, and again at 5- and 10-weeks post-graft.

The drag test measures the ability of the rat to maintain balance during a postural stability challenge [45]. Briefly, the tail was used to gently lift the hind paws approximately 2 inches above the counter surface. The rat was then gently pulled backward over a fixed distance of 1 meter at a constant speed (~1 m/s) while another investigator filmed forepaw step adjusting (i.e., tapping) behavior. A rat with a nigrostriatal lesion will typically drag the affected forepaw across the counter rather than make adjusting steps. Each test day consisted of 3 trials per animal and data are represented as the average number of forepaw taps across the trials for each forepaw.

The sticky paws test is a tactile stimulation task that is sensitive to nigrostriatal damage and used to quantify somatosensory impairment in 6-OHDA-lesioned rats [45, 46]. An adhesive sticker (1cm x 2cm; Avery Products Corp) was placed on each forepaw. The rat was immediately placed back in its home cage and the time recorded for successful removal of both stickers, or two minutes elapsed, whichever occurred first. Latency to sticker removal and forepaw preference were recorded for each trial. Data are represented as the average total latency to successfully remove the stickers (in seconds) across two trials at each time point. All behavior tests in this study were rated and analyzed by investigators blinded to experimental treatment.

**Levodopa Treatment**

Beginning 4 weeks after 6-OHDA surgery, rats were primed for 5 weeks with once daily (M-Fr) injections of levodopa plus benserazide (12 mg/kg, 1:1; subcutaneous (s.c.)). Levodopa was withdrawn for one week following graft surgeries to prevent any potential toxic interaction of the drug with grafted cells [35, 47] and was then reinitiated and continued daily (M-Fr) at this same dose throughout the remainder of the experiment.

**Levodopa-induced Dyskinesia Ratings**

In the current study, we employed the well-validated rat model of LID as our primary indicator of graft function because this complex behavioral malady can be ameliorated by DA neuron grafts in parkinsonian rats [48-52] and individuals with PD (for review [11]). Dyskinetic behaviors were rated according to an LID rating scale developed in our lab in conjunction with a clinical movement disorders specialist (RK; [53, 54]). LIDs were evaluated on pre-graft days 1, 6, 12, 20, and 26, and post-engraftment at weeks 3, 4, 5, 7, 9, and 10. LID were assessed in one-minute intervals at 20, 70-, 120, 170, and 220 minutes following levodopa administration. For each rating session, a "total" LID severity score was calculated for each subject as previously detailed [54]. All rats were rated by the same blinded investigator throughout the duration of the experiment.

**Preparation of Donor Tissue**

VM tissue containing developing A8–A10 DA cell groups were dissected from WT (Val/Val) embryonic day 14 (E14) Sprague-Dawley rat pups (CD® International Genetic Standardization Program; crown-rump length 11-13mm). VM tissue was collected and pooled from multiple WT donors in cold calcium–magnesium free (CMF) buffer, then immediately dissociated and resuspended in Neurobasal™ medium per [16]. Cell number and viability were estimated using trypan blue exclusion. Cells were kept on wet ice during transplantation surgery and used within 4 h of preparation. Cell-free Neurobasal™ medium was used for sham control surgeries.

**Cell Transplantation**

After 5 weeks of levodopa priming, rats were assigned to DA graft and sham graft groups such that the mean pre-graft LID severity scores were statistically similar between groups. Transplantation was carried out as described [16], with modification of engrafted cell number. Briefly, cells were deposited at a single rostral-caudal striatal site (0.2 mm anterior, 3.0 mm lateral, relative to bregma), distributed along three dorsal-ventral coordinates at this site corresponding to 5.7-, 5.0-, and 4.3-mm ventral to the bottom edge of the skull. Rats assigned to the DA-graft group received an intrastriatal transplant of 400,000 VM cells from E14 WT donors. Each dorsal-ventral coordinate received 2 µl (0.5 µl/min) of the VM cell suspension for a total volume of 6 µl per [16]. This VM cell concentration was twice that previously employed in young rats [16] to account for decreased viability of grafted VM neurons in middle-aged rats (~50% reduction) [16, 35, 55]. Sham-grafted rats received a total of 6 µl of cell-free vehicle using the same stereotaxic coordinates.

**Graft-induced Dyskinesia Ratings**

Amphetamine-mediated GID capitalizes on the finding that DA-grafted, but not sham grafted rats display dyskinetic behavior with graft maturation in response to low-dose amphetamine, which appear phenotypically similar to LID [56-59]. Given that GID develop with graft maturation [16, 49]), GID were evaluated 10 weeks post-engraftment/ 24 hours prior to sacrifice. Rats received a single intraperitoneal (i.p.) dose of amphetamine sulfate (2 mg/kg) and were returned to the home cage for behavioral evaluation by a blinded investigator using the same method and rating scale described **Error! Reference source not found.** for LID rating per [16].

**Euthanasia and Post-Mortem Analyses**

Rats were sacrificed as previously detailed [16]. Briefly, rats were deeply anesthetized with phenytoin/pentobarbital euthanasia solution (VetOne, Boise, ID, USA) followed by intracardiac perfusion with 4% paraformaldehyde. Prior to perfusion, an ear clipping was collected from each subject and stored at -80°C for automated genotype confirmation (Transnetyx Inc., Cordova, TN, USA). Brains were post-fixed for 24 hours at 4°C, submersed in 30% sucrose solution, and stored at 4°C until time of sectioning. They were sectioned coronally at 40-µm and stored at -20°C in a cryoprotectant solution. Detailed descriptions of immunohistochemical (IHC) and fluorescent *in situ* hybridization (fISH) methods, stereological quantification of graft cell number, graft volume, graft-derived neurite outgrowth, and fluorescent image acquisition and analyses are as previously detailed [16].

**Statistical Analysis**

All LID and GID data were analyzed with non-parametric statistics including Mann-Whitney U-test (for between-subject contrasts) and Friedman or Kruskal-Wallis with Dunn’s multiple comparison test (for within subjects tests). All parametric data were analyzed by t-tests or multifactorial ANOVAs with appropriate post-hoc analyses after testing for normal distribution. Statistics were completed using GraphPad Prism® software for Windows (v. 10.0.2); additional statistical information is provided in figure legends.

## RESULTS

### Maintenance of the rs6265 SNP enhanced functional benefit in aging DA-grafted rats

Prior to grafting, LID severity during levodopa priming was similar between genotypes (**Figure 2a*,*** *Day of LD Treatment 1-26*), contrasting prior clinical evidence that Met allele carriers with PD tend to develop LID earlier than individuals with the wild-type (WT; Val/Val) genotype [60]. Post-grafting, middle-aged **Met/Met** rats engrafted with WT VM cells demonstrated rapid LID amelioration in comparison to sham-grafted rats (**Figure 2a,d**), demonstrating significant benefit at the first 3-week post-graft timepoint. In contrast, the **WT** rats engrafted with WT VM cells showed no graft efficacy at this timepoint (**Figure 2a,b,c**) requiring an additional 7 days to reveal any graft-derived benefit, not showing significant graft efficacy until week 4 post-grafting. Both DA-grafted groups maintained a robust and similar level of behavioral recovery at week 4 and throughout the remainder of the experiment (**Figure 2a,** *weeks post- graft*).

While there was evidence of maintained functional benefit in Met/Met middle-age rats (15mos at grafting), LID severity results differ from what we previously reported in younger rats (8mos at grafting). Specifically, younger Met/Met rats not only a more rapid reduction in LID, but the overall graft-derived benefit was greater for the entire post-graft time compared to their WT counterparts ([16], reproduced with permission, **Figure 2e,** *week post-graft*). Interestingly, the final DA graft-derived benefit in middle-aged rats in response to engraftment of 400,000 VM cells was a reduction of LID severity (from baseline) by 70.31 ± 9.89% in Met/Met and 66.99 ± 12.71% in WT rats (mean ± SEM). In contrast, the reduction of LID in younger rats was 55.21 ± 2.22% in WT and 73.92 ± 12.51% in Met/Met rats in response to engraftment of *half this number of VM cells* (i.e., 200,000). The number of VM cells engrafted was varied between age groups with the goal of obtaining equal final numbers of surviving grafted DA neurons in young and middle-aged rats based findings of reduced viability of primary DA neurons in aging rats [61]. Counter to previous data demonstrating reduced survival of grafted DA neurons with advancing age, the current data demonstrate that in both genotypes there were at least twice as many surviving grafted DA neurons in middle-aged compared to young subjects (**Figure 3c**). Despite an overall increase in final number of surviving DA neurons in aging parkinsonian rats, when represented as "percent improvement per grafted neuron", young subjects showed significantly greater improvement compared to the middle-aged cohort at the final post-grafting timepoint (**Figure 2f**). There was no effect of genotype on this outcome measure.

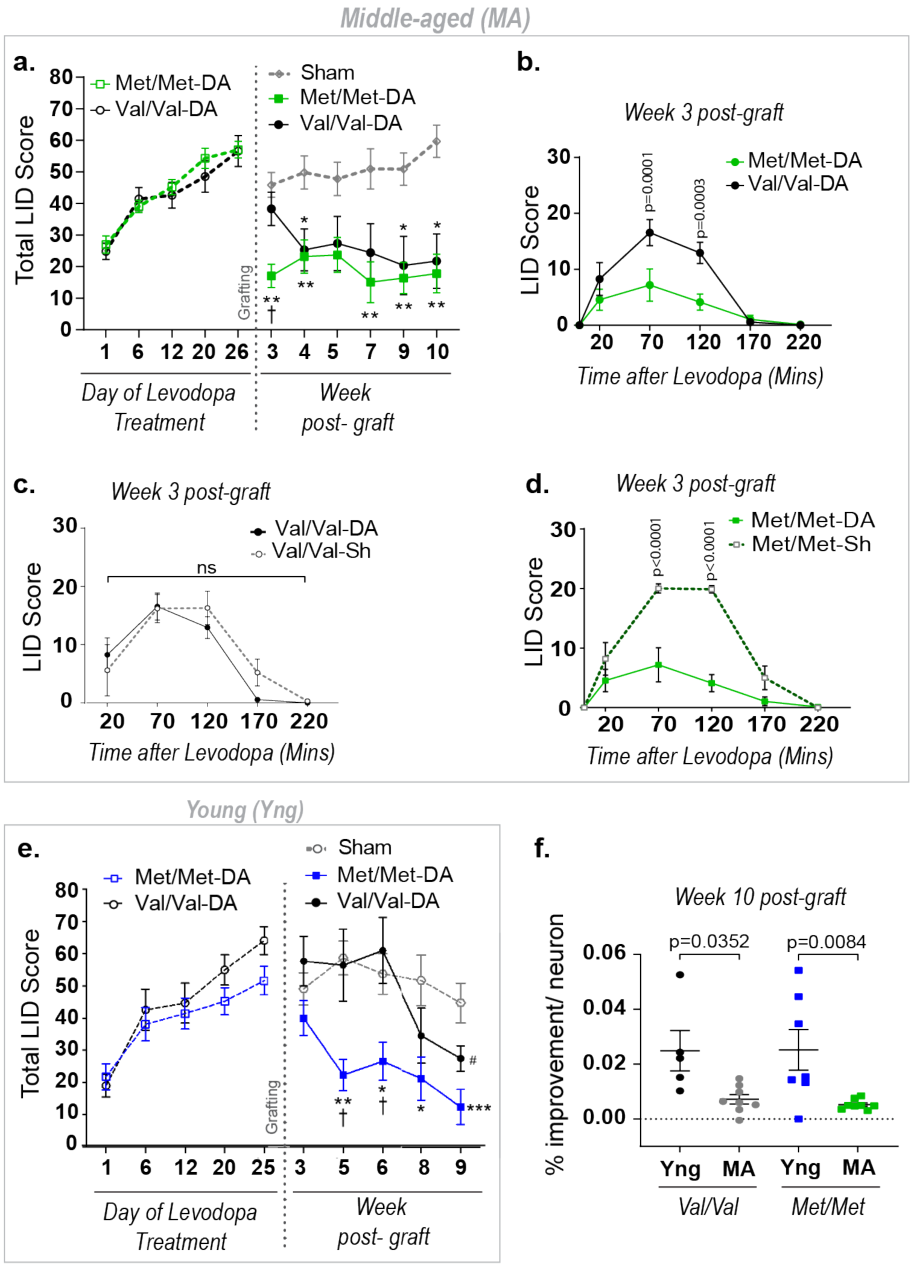

**Figure 2. Impact of host genotype on LID amelioration in middle-aged, parkinsonian rats (a)** Time course of total LID severity throughout the duration of experiment including pre-graft LID priming and post-graft behavioral recovery in Val/Val (WT) and Met/Met hosts. Dyskinesia severity scores from sham grafted animals did not differ between genotypes, therefore data for Val/Val (WT) and Met/Met sham subjects were combined into one group for all post-graft time points. *Statistics:* Kruskal-Wallis test with Dunn's multiple comparisons test at each time point. Week 3: **p=0.0013 Met/Met-DA vs Sham; †p=0.0599 Met/Met-DA vs Val/Val-DA; Wk4: *p=0.0369 Val/Val-DA vs Sham; **p=0.0176 Met/Met-DA vs Sham; Wk5: not significant (ns) Wk7: ns; **p=0.0069 Met/Met-DA vs Sham; Wk9: *p=0.0151 Val/Val-DA vs Sham; **p=0.0079 Met/Met-DA vs Sham; Wk10: *p=0.0076 Val/Val-DA vs Sham; **p=0.0017 Met/Met-DA vs Sham **(b)** Time course of dyskinesia severity over the 220-minute rating period for Week 3 demonstrating the impact of genotype in DA-grafted vs. Sham-grafted middle-aged rats. Figure (c) and (d) demonstrate the differences between sham-grafted and DA-grafted WT (Val/Val) **(c)** and Met/Met **(d)** animals. *Statistics:* Two-way ANOVA with Tukey's multiple comparisons test at each time point: p<0.05 in figure. **(e)** Total LID score for young (Val/Val and Met/Met rats throughout levodopa priming (pre-graft period) and for 9 weeks post-engraftment. Statistics: Non-parametric Kruskal-Wallis test with Dunn's multiple comparisons test at each time point. Week 5: **p = 0.0015 Met68Met-DA vs Sham, †p = 0.0302 Met68Met-DA vs Val68Val-DA; Week 6: *p = 0.0284 Met68Met-DA vs Sham, †p = 0.0475 Met68Met-DA vs Val68Val-DA; Week 8: *p = 0.0198 Met68Met-DA vs Sham; Week 9: ***p = 0.0003 Met68Met-DA vs Sham. **(f)** LID improvement per grafted DA neuron compared between middle-age groups (MA) vs. the young ('Yng') cohort of rats (young data *reproduced with permission from* [16]). *Statistics:* two-way ANOVA; Šídák's multiple comparisons test: p<0.05 in figure. Data are expressed as mean ± SEM.

Despite a genotype-specific impact of DA grafts on LID reduction at week 3 post-grafting, two "OFF medication" sensorimotor function tests did not reveal significant efficacy of rs6265 in aging rats (**Suppl Figure 1**) likely due to the implementation of testing after week 4 post-grafting. These drug-free tests did however provide insight into their utility in preclinical grafting models. First, in the sticky paws task, 6-OHDA-lesioned rats of both genotypes showed significant impairment of forepaw sensorimotor function contralateral to the lesioned striatum as evidenced by an increase in latency to successfully remove the adhesive label from the affected forepaw (**Suppl Figure 1a**). At 5 weeks post-grafting, DA-grafted Met/Met rats, but not WT rats, showed a trend toward improvement in latency to remove the adhesive label from the affected forepaw vs baseline (**Suppl Figure 1b**). Both genotypes showed significant improvement from baseline at 10 weeks post-grafting (**Suppl Figure 1c**) validating the utility of this “OFF medication” behavioral task for evaluating DA graft function.

In the drag test, 6-OHDA-lesioned rats showed a significant impairment of the contralateral forepaw as evidenced by a reduced postural adjustment tapping response (**Suppl Figure 1d**). For this postural stability task, there was no effect of a DA graft on the affected paw for either genotype at any time post-grafting (**Suppl Figure 1e,** *Wk5* post-graft; **Suppl Figure 1f,** *Wk10* post-graft). These findings are not surprising given that DA replacement therapies generally have limited effects on postural instability in PD (e.g., [62]).

**No enhancement of neurite outgrowth in rs6265 middle-aged hosts**

Similar to what we observed previously in young rats [16], the numbers of surviving DA neurons and graft volumes were similar between genotypes in the middle-aged hosts (**Figure 3c,d**). However, in the older middle-aged rats, neurite outgrowth and density were indistinguishable between Met/Met and WT animals (**Figure 3a,b,e**). This is in contrast to the enhanced neurite outgrowth observed at sites distal to the graft in the younger Met/Met compared to WT hosts despite similar numbers of surviving DA neurons (**Figure 3c,e**). Middle-aged rats of both genotypes also demonstrated equivalent total volume of neurite density (**Figure 3e**) as well as “neurite density per graft cell number” (**Figure 3f,g**). The neurite density per grafted cells in the middle-aged rats was significantly less than that seen in young rats, both proximal and distal to the graft (**Figure 3e,f**). It is reasonable to speculate that in the middle-aged rats, the greater number of engrafted and surviving DA neurons could have precluded our ability to detect genotype-specific functional efficacy at later timepoints post-grafting.

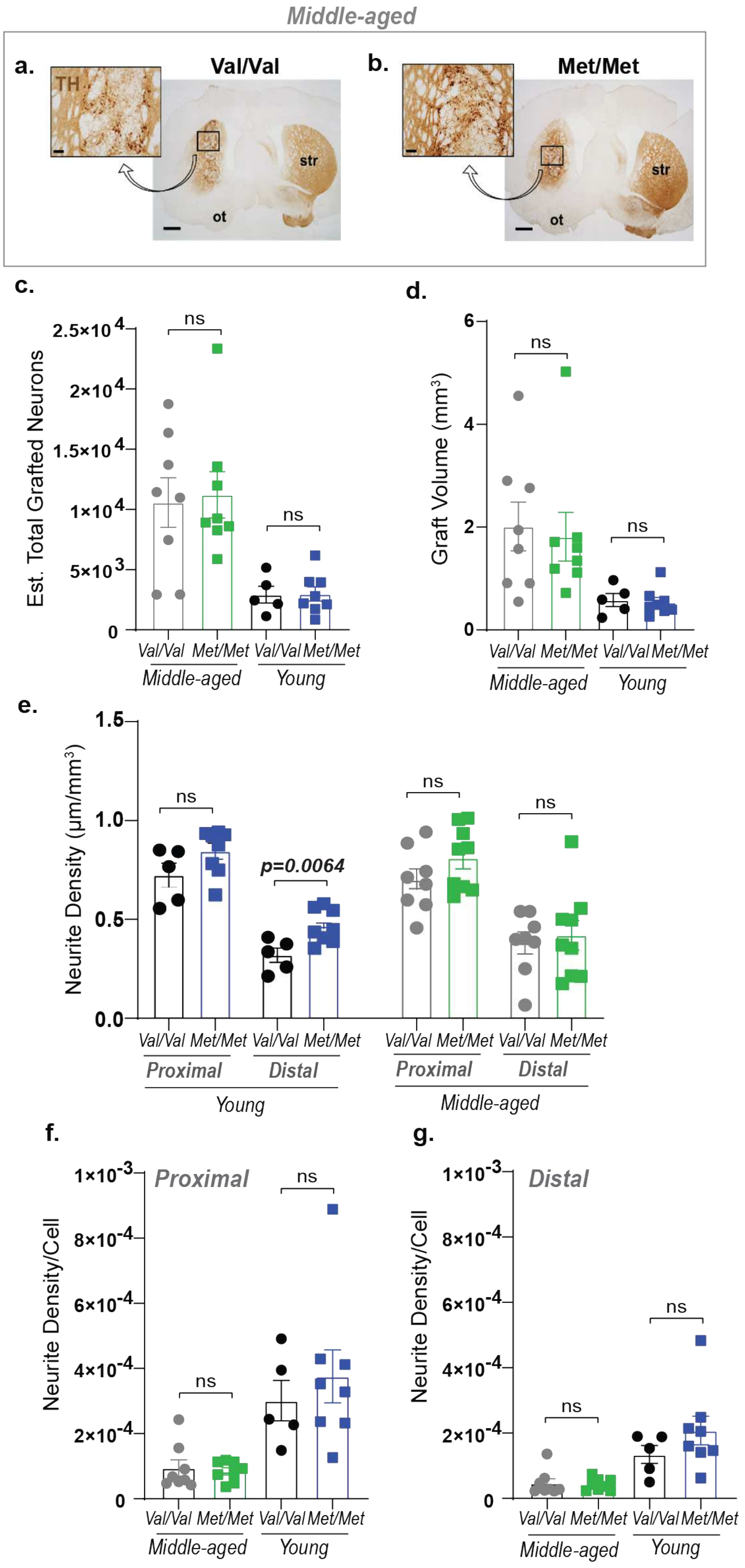

**Figure 3. Impact of host age and genotype on grafted DA neuron survival and neurite outgrowth.** (**a, b**) Representative micrographs of tyrosine hydroxylase immunoreactive (THir) staining in grafted striatum of middle-aged Val/Val (WT) (**a**) and Met/Met (**b**) rats. Scale bars = 1000 µm (1x striatum micrographs), 100 µm (10x micrographs). ot, olfactory tubercle; str, striatum. (**c**) Stereological estimate of total number of surviving grafted THir neurons. *Statistics*: two-way ANOVA (genotype) F (1, 25) = 0.04640, p=0.8312. Tukey's multiple comparison test (ns) .(**d**) Estimated graft volume. *Statistics:* two-way ANOVA (genotype) F (1, 24) = 1.406, p=0.2474. Tukey's multiple comparison test (ns) (**e**) Comparison of total volume of 'neurite density' in both middle-aged and young rats in proximal and distal regions of the grafted striatum. *Statistics:* two-way ANOVA with Tukey's multiple comparison test: p<0.05 in figure. (**f, g**) Comparison of neurite density per grafted cell in proximal (f) and distal (g) regions of the grafted striatum. *Statistics:* two-way ANOVA with Tukey's multiple comparison test (ns).*Young data used with permission from the publisher* [16]. Data are expressed as mean ± SEM

**Increased graft-host appositions correlate with higher LID scores in the aging Met/Met host**

In the current experiment, quantification of the apposition density between grafted TH+ neurites and host synaptopodin (SP), an actin-associated structural protein found in mature dendritic spines ([63, 64]) (TH-SP) (i.e., SP puncta ≤0.6 μm from a TH-immunoreactive (TH-ir) neurite) (**Figure 4a**) revealed that, distal to the lateral edge of the engrafted cells, the Met/Met host striatum contained more TH-SP appositions than observed in the same region of the DA-grafted striatum of Val/Val rats (**Figure 4b,** *distal*). This contrasts the intact contralateral striatum in which the endogenous TH-SP contact density was not different between genotypes (**Figure 4b***, contralateral)*. Importantly, TH-SP connectivity distal to the lateral edge of the graft was significantly associated with *increased LID* severity in Met/Met, but not Val/Val host rats (**Figure 4d**), suggesting that aging may interact with this SNP to alter graft function by promoting the establishment of *abnormal* synaptic connections. Though positive trends were observed for both genotypes proximal to the graft, these correlations were not significant (**Figure 4c**).

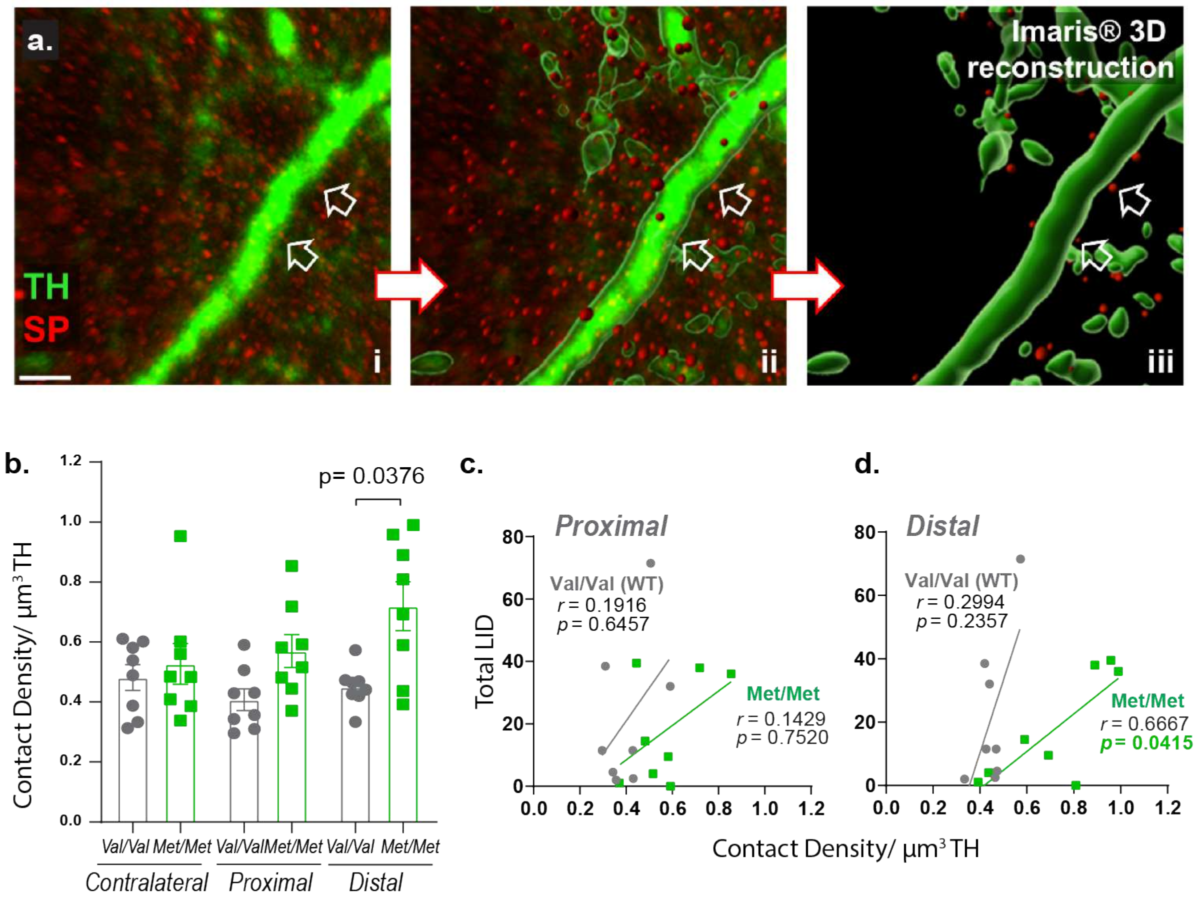

**Figure 4. Impact of host age and genotype on graft-derived neurite appositions with host dendritic spines in the parkinsonian striatum of middle-aged rats. (a)** Representative micrograph of immunofluorescent staining for tyrosine hydroxylase (TH, green) and synaptopodin (SP, red puncta) demonstrating appositions of grafted DA neurites and host striatal dendritic spines, respectively. A confocal micrograph is depicted in (**i**), with two different views of the Imaris® 3D reconstruction presented in (**ii**) and (**iii**). Scale bar = 2 μm. **(b)** Quantitation of TH-SP contact densities in striatum both proximal and distal to the graft, and in the intact contralateral striatum. *Statistics: (Proximal and Distal;* two-way ANOVA with Tukey's multiple comparisons test: $p<0.05$ in figure. (*Contralateral;* two-way ANOVA $p>0.9999$). Error bars are expressed as mean ± SEM. (**c, d**) Spearman correlations of TH-SP contact density and total LID score at the final post-graft behavioral rating time point in middle-aged cohorts. Correlations are shown in both proximal (**c**) (*Statistics: V*al/Val (WT): Spearman $r=0.1916$, $p=0.6457$; Met/Met: Spearman $r=0.1429$, $p=0.7520$) and distal regions (**d**) (*Statistics*: Val/Val (WT): Spearman $r=0.2994$, $p=0.2357$; Met/Met: Spearman $r=0.6667$, $p=0.0415$, bold green).

## Aging and rs6265 SNP expression are permissive to GID development

The current studies demonstrate that all DA-grafted, middle-aged rats exhibited GID behaviors in response to amphetamine *regardless of host genotype*. Sham-grafted subjects showed no appreciable GID behavior (**Figure 5a,b**). While there was an emergence of GID in both genotypes, peak severity scores were significantly greater in the Met/Met compared to WT rats, both engrafted with WT DA neurons (**Figure 5a,** *DA-graft*). GID severity in the Met/Met hosts also showed a protracted increase in severity between the 20 and120 minute rating times compared to sham rats or DA-grafted WT rats (**Figure 5b**), again suggesting that aging interacts with this SNP to alter graft function in Met allele carriers.

These findings in aging subjects are distinct from what we previously observed in young rats, where only Met/Met subjects developed GID in the presence of a large DA graft with widespread reinnervation (**Figure 5c**, *DA-graft, reproduced with permission* [16]). Young WT rats with a widespread intrastriatal DA-graft did not develop GID [16], unlike what was observed in middle-aged DA-grafted WT rats (**Figure 5a**, *DA-graft*). Further, middle-aged rats and/or those expressing the Met/Met genotype present not only with increased severity of GID but also with an increase GID incidence (absolute peak score of $\geq$5) compared to all other experimental groups (**Figure 5d**).

## Grafted DA neurons maintain an immature phenotype in aging hosts

During development, nigral DA neurons express vesicular glutamate transporter 2 (*Vglut2)* mRNA and VGLUT2 protein, showing evidence of DA-glutamate co-transmission; however, this phenotype typically disappears with neuronal maturation (for review [65]). We previously demonstrated that this signature of immature DA-glutamate co-transmission is maintained in grafted DA neurons regardless of host genotype, but that it was significantly correlated with GID only in young Met/Met rats [16]. Here we demonstrate that grafted DA neurons in middle-aged rats also contain *Vglut2* mRNA (**Figure**

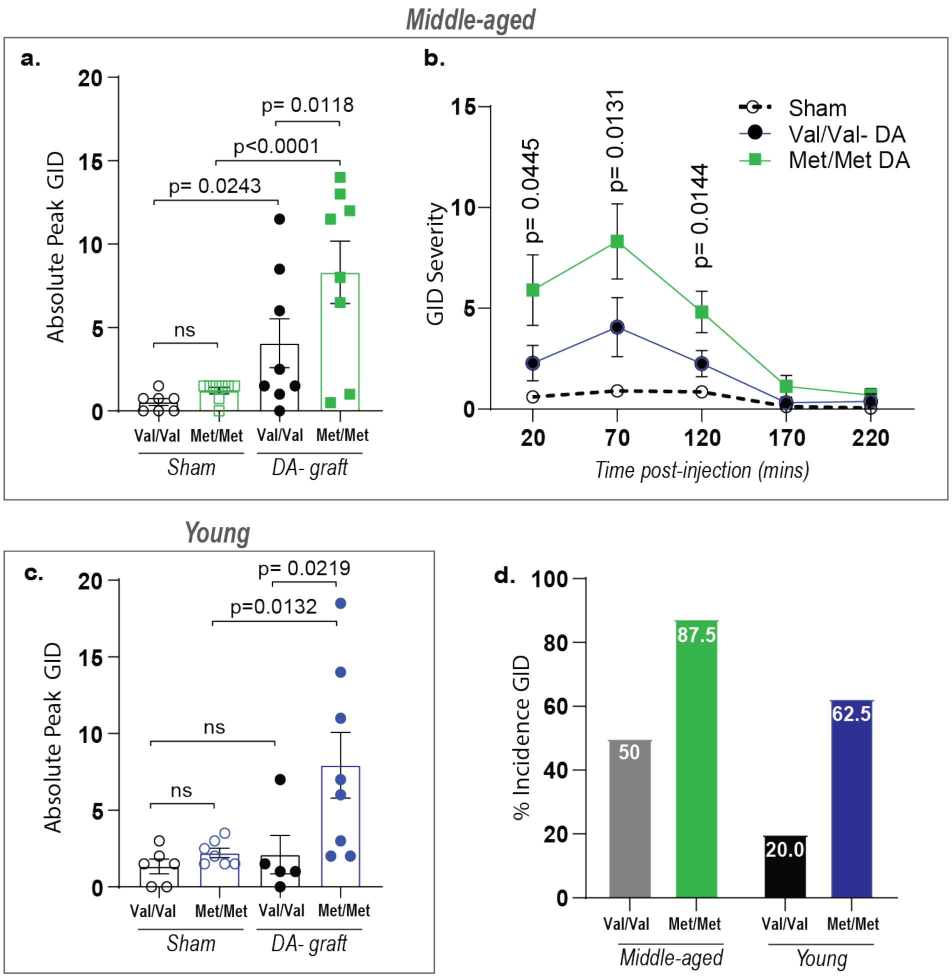

**Figure 5. Impact of host age and genotype on graft-induced dyskinesias (GID)** (**a**) Graph of absolute peak GID severity at 10 weeks post-engraftment for middle-aged rats. *Statistics:* two-way ANOVA with Tukey's multiple comparisons test: p<0.05 in figure. (**b**) Time course of GID at 10 weeks post-engraftment. All animals were rated in one-minute intervals at 20, 70, 120, 170, and 220-minutes post injection. *Statistics:* two-way ANOVA with Tukey's multiple comparisons test: p<0.05 in figure. (**c**) Absolute peak amphetamine-mediated GID severity at 10 weeks post-engraftment for young rats' injection. *Statistics:* two-way ANOVA with Šídák's multiple comparisons test p<0.05 in figure. (**d**) Percent incidence of middle-aged rats in each group with an absolute peak GID score of 5 or higher. *Young data used with permission from the publisher* [16]. Data are expressed as mean ± SEM.

**6a,b**) and that levels are equivalent between aging Met/Met and Val/Val rats. Interestingly, *Vglut2* mRNA levels were lower in grafted DA neurons of the middle-aged rats compared to younger rats (**Figure 6b**) despite the absolute percentage of grafted DA neurons positive for *Vglut2* mRNA expression not differing between genotypes, or between young and middle-aged graft recipients (**Figure 6c**).

Similar to what was observed in younger rats, VGLUT2 protein was detected *within THir neurites* in the parkinsonian striatum (**Figure 6d**). In the middle-aged rats, no significant differences were observed between genotypes comparing naïve intact (non-lesioned) and grafted striatal hemispheres (**Figure 6e**). This contrasts findings in younger subjects where there was significantly more VGLUT2-TH colocalization in the contralateral intact and DA-grafted striatum of parkinsonian rats compared to the naïve striatum (**Figure 6f,g,** *reproduced with permission* [16]). Of note, despite the increase in GID in DA-grafted middle-aged subjects of both genotypes, there was no correlation between

VGLUT2 protein expression within TH+ neurites and GID severity in either middle-aged genotype (**Figure 6h)**. These results strikingly contrast what was reported in the younger cohort [16] where

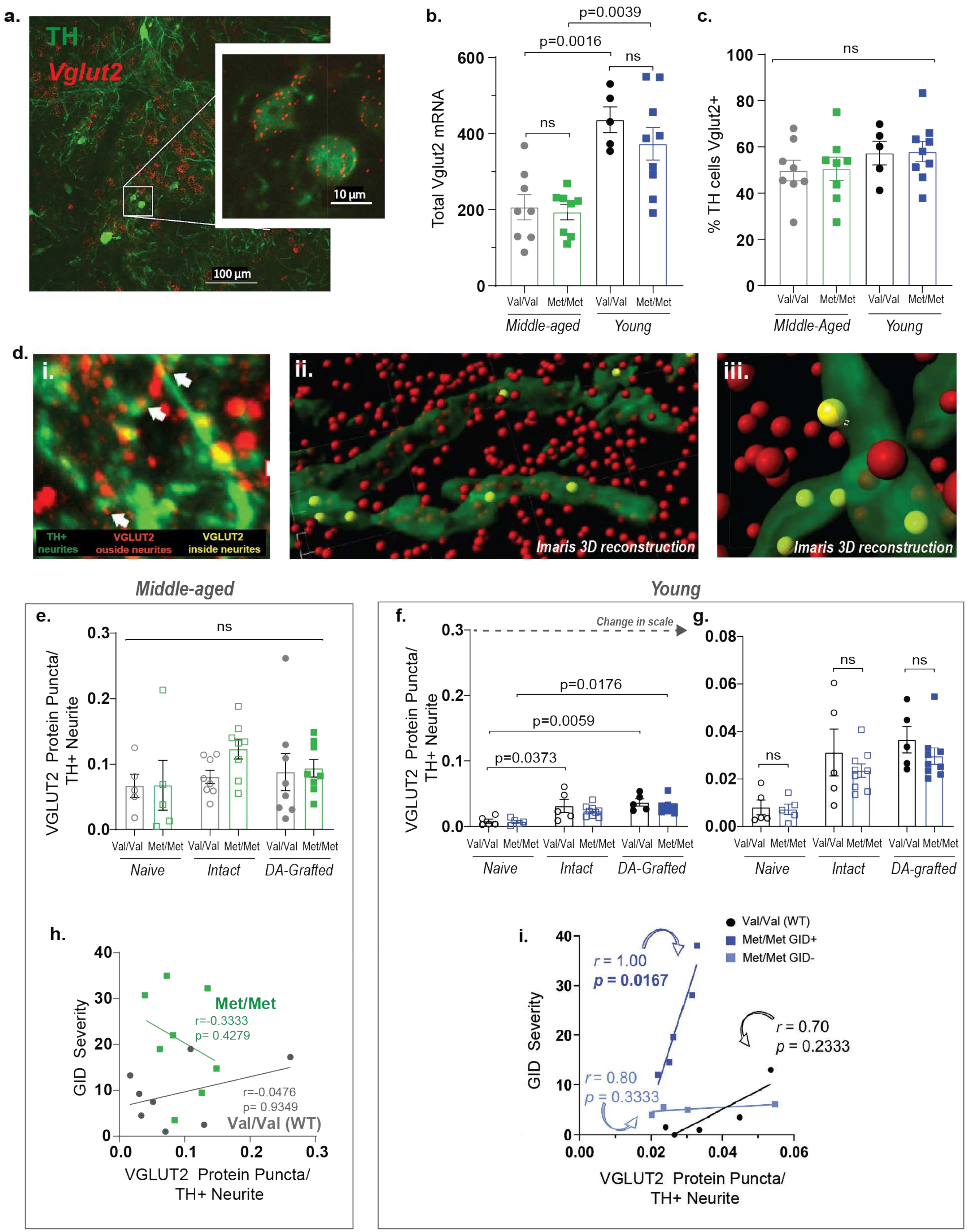

**Figure 6. Impact of host age and genotype on Vglut2 mRNA and protein expression in grafted DA neurons: Relationship to GID.** (**a**) Representative confocal micrograph depicting dual label staining for TH protein and Vglut2 mRNA in grafted DA neurons. Scale bars = 100 µm and 10 µm (inset). (**b, c**) Comparison of total Vglut2 mRNA present within the graft (combined TH+/Vglut2+ and TH-/Vglut2+ cells), normalized to graft size (i.e., number of DA neurons present in the tissue section) of young and middle-aged Val/Val (WT) and Met/Met rats. *Statistics:* two-way ANOVA with Tukey's multiple comparison test. $p<0.05$ in figure. (**c**) Percentage of DA neurons expressing *Vglut2* mRNA in middle-aged, sham and DA-grafted parkinsonian striatum in young and middle-aged rats. *Statistics:* two-way ANOVA with Tukey's multiple comparison test (ns). (**d**) Visualization of VGLUT2 colocalization within grafted DA fibers, reconstructed in 3D with Imaris®. This reconstruction was created from a representative confocal z-stack of grafted striatal tissue immunolabeled for TH and VGLUT2. Arrows indicate VGLUT2 protein (yellow puncta) contained within grafted DA neurites; red puncta are VGLUT2 external to TH fibers. Scale bar = 1 µm. (**e, f**) Quantification of VGLUT2-TH colocalization, normalized to volume of the TH surface in middle aged (**e**) and young rats (**f**). *Statistics:* two-way ANOVA with Tukey's multiple comparison test: $p<0.05$ in figure. (**g**) Quantification of VGLUT2-TH colocalization in young rats with a modified y-axis scale to better illustrate differences between groups. Tukey's multiple comparison test (ns). (**h, i**) Non-parametric Spearman correlations between amphetamine-mediated GID and VGLUT2-TH colocalization in middle-aged (h) Val/Val: *Statistics:* Spearman $r=0.0476$, $p=0.9349$; Met/Met: Spearman $r=-0.3333$, $p=0.4279$) and young (i) grafted subjects. *Abbreviations*: GID, graft-induced dyskinesias; ns, not significant ($p>0.05$); TH, tyrosine hydroxylase; Vglut2/VGLUT2, vesicular glutamate transporter 2. *All data for the young cohort was used with permission from the publisher* [16].

VGLUT2-TH colocalization was demonstrated to be positively correlated with amphetamine-mediated GID in Met/Met rats [16] (**Figure 6i,** *reproduced with permission* [16]).

### Serotonin (5-HT) neurons are present in DA grafts but not associated with GID

In middle-aged graft recipients, 5-HT neurons were observed in all grafts as marked by tryptophan hydroxylase 2 (*Tph2*; the rate-limiting enzyme in the synthesis of serotonin (5-HT)) mRNA (**Figure 7a**). The number of 5-HT neurons relative to DA neurons (5-HT/DA ratio) did not differ between genotypes in the older animals in contrast to what was observed in the younger cohort where there was a higher ratio of 5-HT to DA neurons in Met/Met compared to WT grafted rats (**Figure 7b,** *young data reproduced with permission* [16].) The 5-HT/DA ratio also did not differ between age groups. Moreover, despite the controversy that 5-HT neurons contribute to GID in DA-grafted PD patients, the 5-HT/DA ratio was not correlated with GID (**Figure 7c**) or LID in the present experiment (**Figure 7d**) or in the young cohort [16], suggesting that there are other underlying mechanisms driving GID development in these animals.

### Striatal D2 DA receptor mRNA expression is not associated with GID

The D2 DA receptor, DRD2, has been implicated in GID pathogenesis in part related to the reduction of this behavior following administration of the DRD2 antagonist buspirone [66, 67]. Therefore, we compared DA receptor D2 subtype *(Drd2)* mRNA in the dorsolateral striatum of DA-grafted subjects in the intact and lesioned striatal hemisphere of young and middle-aged WT and Met/Met rat hosts. (**Figure 7e,h**). The data collected in regions of the lesioned striatum reinnervated by graft-derived THir fibers showed that, in middle-aged hosts, *Drd2* mRNA expression was significantly higher in the DA-depleted striatum of Met/Met compared to WT subjects (**Figure 7e,f**; *Middle-aged*). This genotype comparison was not statistically significant in young rats, albeit the trend follows a similar pattern (**Figure 7f**; *Young)*. There was also no significant correlation between *Drd2* mRNA expression at any age or genotype with GID severity (**Figure 7g,h**) with the middle-aged data suggesting that if there is an association of D2DR with GID, other means of detection (e.g., receptor binding) will be necessary.

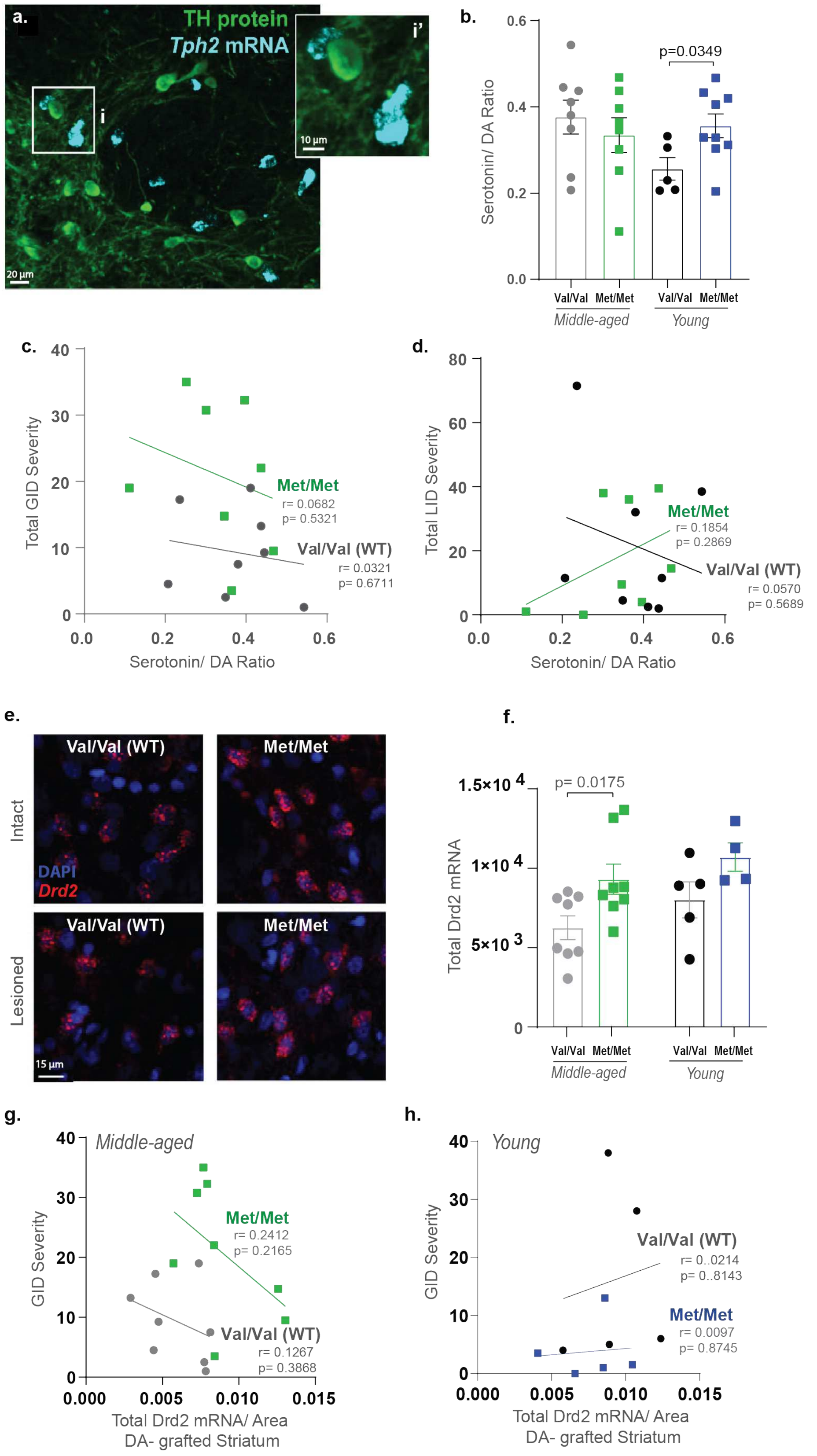

**Figure 7. Impact of host age and genotype on serotonin (5-HT)/DA cell composition and D2 receptor (*Drd2*) mRNA expression in DA grafted subjects. (a)** Representative confocal micrograph showing tryptophan hydroxylase 2 (*Tph2*) (i.e., 5-HT) neurons interspersed among THir DA neurons in the grafted parkinsonian striatum of a middle-aged subject. Box indicated by (i) is shown with increased magnification in (i'). Scale bars = 20 µm and 10 µm (inset). **(b)** Quantification of 5-HT neurons relative to the number of DA neurons within grafted striatum. Mean ± SEM. Two-way ANOVA with Šídák's post-hoc test (Middle-aged vs. Young (ns); Val/Val (WT) vs Met/Met p=0.0349; Young). *Young cohort data used with permission from the publisher* [16]. **(c, d)** Non-parametric Spearman correlations between 5-HT/DA ratio and GID **(c)** and LID in middle-age rat hosts **(d)**. **(e)** Representative confocal micrographs depicting *Drd2* mRNA (red) in dorsolateral striatum of middle-age rats and counterstained with the nuclear DNA stain DAPI (blue) from DA-grafted subjects, in both lesioned and intact striatum. Scale bar = 15 µm. **(g)** Quantification of total *Drd2* mRNA in middle-age and young DA-grafted subjects. Two-way ANOVA (Met/Met vs Val/Val (WT); age (ns) with Šídák's multiple comparisons test. (Middle-aged vs. Young (ns); Val/Val (WT) vs Met/Met p=0.0175; Middle-age). **(h, i)** Non-parametric Spearman correlations between total striatal *Drd2* mRNA per area and GID in Middle-aged (h) and young (i) rat hosts (ns, statistics in graph). *Abbreviations:* DA, dopamine; Drd2, dopamine receptor D2; GID, graft-induced dyskinesias; LID, levodopa-induced dyskinesias.

## DISCUSSION

Recent years have seen a rapid resurgence in clinical trials for the experimental therapeutic approach of cell transplantation for PD. While steps have been taken to optimize patient selection and cell transplantation methods, several noteworthy issues remain to be resolved. Foremost, there remains a lack of understanding of mechanisms underlying GID and why some graft recipients experience little or no clinical benefit despite robust survival of grafted DA neurons (e.g., [68, 69]). Unknown factors within the aged parkinsonian brain continue to limit the applicability of this experimental approach to the elderly populations [8, 35]. Despite intense interest in understanding the role of genetics in the pathogenesis of PD (for review [70]), the impact of specific genetic risk factors has yet to be considered in therapeutic responsiveness to neural grafting in PD. We recently provided the first preclinical evidence that a common human SNP (i.e., rs6265), known to impact PD patients' response to levodopa [71, 72], also significantly impacts graft-mediated behaviors in young adult parkinsonian rats [16]. Specifically, young rats homozygous for this genetic variant (i.e., Met/Met) displayed a paradoxical enhancement of graft efficacy despite diminished extracellular BDNF in the host striatum [16]. These subjects also uniquely developed GID despite robust widespread graft-derived reinnervation; a finding contrary to clinical and preclinical data that has linked smaller, focal "hot-spot" grafts with GID development [6, 49, 73]. Given that aging is an irrevocable process characterized by the progressive deterioration of normal physiological states and is the primary risk factor for PD [74, 75], we critically explored the interaction of aging with rs6265 in the context of grafting in the parkinsonian brain. We provide here the first preclinical evidence demonstrating that there remains a paradoxical enhancement of graft-derived benefit associated with this common human SNP in aging Met/Met hosts, and for the first time demonstrate that the aging brain is permissive of GID expression with rs6265 further exacerbating this aberrant behavioral profile.

### The rs6265 SNP maintains graft benefit into middle age

It is well established in both clinical trials (e.g., [6]) and animal models (e.g., [8, 35]) that advancing age diminishes benefit of DA cell replacement therapy. While the current study continues to support this tenant, there is indication that the rs6265 genotype in the parkinsonian host maintains capacity to provide therapeutic enhancement in subjects of advancing age following engraftment of WT DA neurons. Specifically, we provide evidence that DA-grafted middle-aged Met/Met rats exhibit earlier, more robust reversal of LID compared to WT rats; however, this genotype-specific benefit was transient, demonstrating a loss of differential graft-derived efficacy between genotypes beginning four weeks post-engraftment. Despite this transitory evidence of rs6265-derived benefit in aging rats, the overall “percent behavioral improvement per neuron” as well as “neurite density per grafted cell” was significantly less in middle-aged compared to young rats regardless of genotype -- this despite twice as many VM cells engrafted into the middle-aged hosts.

In both young and middle-aged parkinsonian rats, the relative degree of neurite outgrowth from engrafted DA neurons correlates with behavioral efficacy. While neurite outgrowth and behavioral benefit was significantly greater in young Met/Met compared to WT rats [16], the current study demonstrates that the impact of the Met risk allele on this anatomical correlate was not maintained even just into middle-age. Despite more rapid graft benefit in middle-aged Met/Met subjects than in WT, the current data revealed similar levels of final neurite outgrowth at sacrifice, which was mirrored by similar levels of final behavioral efficacy in both cohorts. This evidence could be taken to suggest that the variant rs6265 genotype in graft recipients encourages *faster* neurite outgrowth from grafted DA neurons at earlier time points post-engraftment, and as grafts mature, slower neurite outgrowth in WT graft recipients eventually reaches the same density as that of transplanted cells in Met/Met hosts under our experimental parameters. Future studies employing less abundant graft cell density will be needed to determine definitively whether the Met allele is capable of conferring neurite outgrowth enhancement in the aging brain, a scenario that could be of translational significance in cases of inferior survival of transplanted neurons.

While the discovery of enhanced therapeutic efficacy and neurite outgrowth in parkinsonian rats homozygous for the Met allele seemed paradoxical to what would be expected in an environment of diminished BDNF availability (e.g., [32]), it is important to realize that the rs6265 risk allele has previously been implicated in enhanced recovery in a rs6265 transgenic mouse stroke model [76] and enhanced recovery of executive functioning in Vietnam veteran Met allele carriers who had sustained combat-related traumatic brain injury (TBI) [77]. Considering the abundance of the rs6265 Met allele expression in the human population, it would seem illogical that such a common genetic variant would be disadvantageous [78]. Our work, here and previously [16], demonstrate a beneficial role for the variant Met allele in axonal growth and regeneration such that even WT DA neurons can be induced to develop this enhanced phenotype when transplanted ectopically into a Met allele-carrying host. The valine to methionine substitution associated with the rs6265 SNP lies within the pro-domain of BDNF. The pro-domain is cleaved at the N-terminus of the precursor molecule resulting in liberation of the active mature BDNF and the pro-peptide. Intriguingly, the BDNF pro-peptide has been suggested to act as a novel biomolecule, which we posit may be responsible for the beneficial role for the variant Met allele in axonal growth and regeneration [16, 79]. Further investigation into this mechanism could provide a therapeutic means of promoting neuroregeneration in neurodegenerative disease.

**Aging and rs6265 expression increase GID incidence and severity**

Despite the Met allele being associated with enhanced functional benefit in preclinical neural grafting studies, and in other neurologic conditions [76, 77, 80], our preclinical studies continue to support that an individual's genotype (i.e., the rs6265 allele) may drive aberrant mechanisms associated with the GID behavioral phenotype. In addition, there is data suggesting that the aged brain may be primed for amplified GID development associated with dendritic spine loss [79, 81, 82] and impaired synaptic plasticity [83, 84] that are both associated with normal aging. One hypothesis regarding GID pathogenesis is that abnormal patterns of graft-host synaptic connectivity underlie this aberrant graft-induced side-effect [11, 14, 85]. Indeed, abnormal graft-host synaptic integration has been observed in rodent and non-human primate models of PD, as well as in postmortem clinical PD cases [50, 86-88], with these abnormal connections significantly correlated with GID in parkinsonian models [16, 50]. Given that aging-associated synaptic plasticity alterations are correlated with age-related changes in BDNF availability/activity [89, 90], and that extracellular BDNF levels are reduced in association with rs6265, we hypothesized that advancing age would promote development of GID and that the presence of the variant Met allele would exacerbate GID in aged parkinsonian subjects. The current study provides the first evidence that even WT rats of advancing age develop GID despite the presence of widespread DA graft-derived reinnervation, and that in aging Met/Met hosts, this aberrant graft-mediated side effect has a higher incidence of occurrence and is significantly more severe.

**Interaction of host age and genotype on graft-derived reinnervation**

As recently reviewed [91], the exact nature of nigrostriatal dopaminergic synapses has been a matter of long-standing debate. In the normal adult striatum, it has been principally considered that DA is released from varicosities largely devoid of defined synaptic characteristics, although some dopaminergic synapses can be found that exhibit ultrastructural features common to symmetric (Gray type-II/inhibitory) synapses [91-94]. We have continued to examine the nature of graft-host connectivity in our current studies involving middle-aged WT and Met/Met subjects engrafted with primary DA neurons to provide new insight into the complexities of graft reinnervation characteristics and behavioral output. We previously demonstrated that the apposition density between grafted THir neurites and host SP, an actin-associated structural protein found in mature dendritic spines [63, 64] (TH-SP), was significantly correlated with graft efficacy (i.e., decrease in LID severity) in younger WT rats, but not in Met/Met rats [16]. The association suggests that grafted DA neurons in young rats are making physiologically normal synaptic appositions with WT host striatal medium spiny neurons [16, 35].

Strikingly, the results of the current study demonstrated that in middle-aged, DA-grafted Met/Met rats increased TH-SP contact density was significantly correlated with reduced graft function (i.e., less amelioration of LID severity) at the final post-graft behavioral rating. These results suggest that the Met allele and aging exacerbates the development of non-physiological graft-host connectivity, which impairs graft efficacy in elderly subjects carrying the rs6265 BDNF SNP, and/or contribute to the pathological induction of GID. Given that there was an inverse correlation of TH-SP apposition density in middle-aged Met/Met hosts with LID remediation, it is likely that other cellular mechanisms underlie the enhanced behavioral benefit (i.e. reduced LID severity soon after engraftment) in rs6265 rats.

As introduced above, while nigrostriatal dopaminergic neurons typically make <u>sym</u>metrical structural appositions with striatal neurons, atypical **<u>a</u>**<u>sym</u>metric (Gray type-I/*excitatory*) synapses formed by THir fibers have been documented postmortem in the DA-grafted striatum from persons with PD [88], parkinsonian non-human primates [87], and parkinsonian rats [50]. In parkinsonian rats, we previously reported that ultrastructurally defined asymmetric synapses made by grafted DA neurons are associated with GID [50]. More recently, we confirmed for the first time in young Met/Met rats the presence of VGLUT2 within transplanted TH-ir neurites (definitive evidence of DA-glutamate co-transmission) and that this was strongly correlated with GID expression [16]. The abundance of VGLUT2 within THir neurites of the grafted striatum, while atypical in adult SN DA neurons, supports that grafted DA neurons maintain an immature phenotype typically seen during gestational development [65, 88]. The current study supports that many of the grafted DA neurons maintain a VGLUT2+ immature neurotransmitter phenotype in middle-aged rat hosts. However, there was no longer a correlation of this neurochemical signature with GID expression in middle-aged hosts as was seen in young Met/Met animals. These divergent findings suggest that additional factors concomitant to, or perhaps distinct from, VGLUT2 expression in the aged brain contribute to GID expression in aged parkinsonian subjects.

**DA signaling and GID**

Raphe-derived 5-HT neurons are currently considered a source of non-physiological DA signaling that contributes to LID given that they sprout into the denervated striatum and convert levodopa to DA, releasing it as a “false neurotransmitter” in the absence of regulatory mechanisms (e.g., DA auto-receptors) [95]. Additionally, 5-HT neurons have been implicated, with a fair degree of controversy, as a causative factor of GID in grafted PD patients. Similar to findings in clinical grafting trials [96, 97], while there is evidence of 5-HT neurons in our VM grafts, our data do not support any relationship between engrafted 5-HT neurons and GID, or LID, in middle-aged parkinsonian rats of either genotype. Though this topic remains a matter of debate, most available evidence supports a major role for the DA system in the development of GID, with a modulatory role for the 5-HT system [56, 98-100].

Indeed, D2 DA receptors (DRD2) have been implicated in GID pathogenesis based in part on the reduction of this behavior following administration of the DRD2 antagonist buspirone in grafted PD patients [67, 101]. However, its action as a partial agonist at presynaptic 5-HT1A auto-receptors is suggested to also modulate dysregulated DA release from engrafted 5-HT neurons (for review [102]). In the current study, we evaluated striatal *Drd2* mRNA expression in association with GID development in parkinsonian rats as an initial step for evaluating this mechanism in our model. Further, because BDNF signaling mediates DA release [103-108], it is reasonable to suggest that the rs6265 BDNF SNP alters DA release in the striatum. We observed higher levels of *Drd2* mRNA in middle-aged Met/Met compared to WT subjects, with a similar albeit not statistically significant trend in young hosts. Despite these findings, there was an absence of a statistical correlation between *Drd2* mRNA expression and GID behavior. It should be noted that these initial findings do not necessarily imply that DRD2 protein and/or DRD2 binding affinity would not be correlated with GID, and therefore additional research is warranted to determine the extent to which striatal DRD2 contributes to GID pathogenesis.

**Conclusion**

While the current work provides a foundation for additional investigations, it carries important translational implications. These results underscore the importance of considering not only global (e.g., aging) but also specific (e.g., genetics) individual characteristics when prescribing therapies for individuals with PD to ensure the safest, most effective treatment for each patient. As the field of PD

progresses toward precision-based medicine, it will be critical to address how genetic heterogeneity contributes to variable therapeutic outcomes of cell replacement therapy in current and past PD patient populations [6, 14]. Overall, the data presented here strongly support the recommendation that clinical participants should be genotyped for the rs6265 SNP, either during patient selection or during study result interpretation, so that the impact of this common variant in individuals with PD may be evaluated.

**Acknowledgments:** This study was supported by the National Institute of Neurological Disorders and Stroke grant NS105826 (KSC, CES, TJC) and the Michigan Parkinson Foundation (KSC, NMM). The authors have no other conflicts of interest to report. We would like to acknowledge the excellent technical assistance of Brian Daley, BS and Christopher Kemp, MS.

**Data Availability**

The dataset used and/or analyzed during the current study are available after publication from the corresponding author upon reasonable request.

**Supplemental Figure 1. Impact of host genotype on drug-free ("OFF-time") parkinsonian sensorimotor function prior to and following grafting. (a)** The 'sticky paws' tactile stimulation task showing a significant increase in average latency (in seconds) to remove the adhesive label from the affected forepaw contralateral to the lesion at baseline (8 weeks pre-graft) in both Val (Val/Val, WT) and Met (Met/Met) cohorts. *Statistics:* two-way repeated measures ANOVA with Šídák's multiple comparisons test: Val/Val: $t(46)=6.648$, $p<0.0001$, Met68Met: $t(46)=4.814$, $p<0.0001$). **(b)** Average latency to remove the adhesive label from the affected contralateral forepaw at 5 weeks post-graft (post) compared to baseline (pre) was not significantly different in either Val (Val/Val, WT) or Met (Met/Met) subjects. *Statistics:* two-way repeated measures ANOVA with Šídák's multiple comparisons test: Val/Val: $p=0.7519$, Met/Met: $p=0.0673$). **(c)** Average latency to remove the adhesive label from the affected contralateral forepaw at 10 weeks post-graft (post) compared to baseline latency (pre-graft) showed significant improvement in both Val and Met cohorts. *Statistics:* two-way repeated measures ANOVA with Šídák's multiple comparisons test: Val/Val: $p=0.0080$, Met/Met: $p=0.0047$). **(d)** The 'drag test' measure of balance and postural stability demonstrated a significant decrease in the average number of forepaw taps with the affected forepaw contralateral to the lesion at baseline evaluation for both genotypes. Met compared to Val subjects demonstrated significantly more taps in the affected paw. *Statistics:* mixed-effects model with repeated measures with Šídák's multiple comparisons test for left vs right forepaw: Val68Val $t(12)=11.97$, $p<0.0001$, Met68Met $t(12)=9.438$, $p<0.0001$; one statistical outlier was removed from the Val/Val group for the intact forepaw). **(e, f)** There was no apparent graft-derived benefit in restoring postural step adjustment frequency (taps) in the affected paw for either genotype, neither at (**e**) 5 weeks post-graft (pre-vs post-graft, mixed-effects model: $p=0.3566$ nor at (**f**) 10 weeks post-graft (pre-vs post-graft, two-way repeated measures ANOVA: $p=0.4753$. All data are expressed as mean ± SEM. *Abbreviations:* Int, intact; Les, lesioned; ns, not significant ($p>0.05$)
